# Serum STIP1, a Novel Indicator for Microvascular Invasion, Predicts Outcomes and Treatment Response in Hepatocellular Carcinoma

**DOI:** 10.3389/fonc.2020.00511

**Published:** 2020-04-30

**Authors:** Xiao-Lu Ma, Wei-Guo Tang, Min-Jie Yang, Su-Hong Xie, Min-Le Wu, Guo Lin, Ren-Quan Lu

**Affiliations:** ^1^Department of Clinical Laboratory, Shanghai Cancer Center, Fudan University, Shanghai, China; ^2^Department of Oncology, Shanghai Medical School, Fudan University, Shanghai, China; ^3^Department of Liver Surgery, Liver Cancer Institute, Zhongshan Hospital, Fudan University, Shanghai, China; ^4^Department of Hepatobiliary and Pancreatic Surgery, Minhang Hospital, Fudan University, Shanghai, China; ^5^Department of Interventional Radiology, Zhongshan Hospital, Fudan University and Shanghai Institute of Medical Imaging, Shanghai, China; ^6^Department of Clinical Laboratory, Shanghai Public Health Clinical Center, Fudan University, Shanghai, China

**Keywords:** hepatocellular carcinoma, resection, TACE, prognosis, STIP1, serum biomarker, microvascular invasion

## Abstract

**Background:** Previous studies reported that stress-induced phosphoprotein 1 (STIP1) can be secreted by hepatocellular carcinoma (HCC) cells and is increased in the serum of HCC patients. However, the therapy-monitoring and prognostic value of serum STIP1 in HCC remains unclear. Here, we aimed to systemically explore the prognostic significance of serum STIP1 in HCC.

**Methods:** A total of 340 HCC patients were recruited to this study; 161 underwent curative resection and 179 underwent transcatheter arterial chemoembolization (TACE). Serum STIP1 was detected by enzyme-linked immunosorbent assay (ELISA). Optimal cutoff values for serum STIP1 in resection and TACE groups were determined by receiver operating characteristic (ROC) analysis. Prognostic value was assessed by Kaplan-Meier, log-rank, and Cox regression analyses. Predictive values of STIP1 for objective response (OR) to TACE and MVI were evaluated by ROC curves and logistic regression.

**Results:** Serum STIP1 was significantly increased in HCC patients when compared with chronic hepatitis B patients or health donors (both *P* < 0.05). Optimal cutoff values for STIP1 in resection and TACE groups were 83.43 and 112.06 ng/ml, respectively. High pretreatment STIP1 was identified as an independent prognosticator. Dynamic changes in high STIP1 status were significantly associated with long-term prognosis, regardless of treatment approaches. Moreover, post-TACE STIP1 was identified as an independent predictor for OR, with a higher area under ROC curve (AUC-ROC) than other clinicopathological features. Specifically, pretreatment STIP1 was significantly increased in patients with microvascular invasion (MVI), and was confirmed as a novel, powerful predictor for MVI.

**Conclusions:** Serum STIP1 is a promising biomarker for outcome evaluation, therapeutic response assessment, and MVI prediction in HCC. Integration serum STIP1 detection into HCC management might facilitate early clinical decision making to improve the prognosis of HCC.

## Introduction

Hepatocellular carcinoma (HCC) is one of the most prevalent malignancies worldwide, with increasing incidence and mortality rates (1–3). Radical resection is considered the only curative approach for early-stage HCC. However, despite impressive innovations in surgical procedures, the overall survival (OS) of HCC patients remains unsatisfactory due to high incidence of recurrence or relapse after surgery (4–6). Nonetheless, for irresectable, intermediate-HCC patients, transcatheter arterial chemoembolization (TACE) has evolved as a recommended approach by the Barcelona Clinic Liver Cancer (BCLC) criteria to achieve stable clinical benefit in these patients (7, 8). Unfortunately, response rates are dramatically heterogeneous, and long-term prognosis also remains poor after TACE (9, 10). Therefore, identification of a reliable biomarker to assist pre-interventional stratification is urgently required.

Stress-induced phosphoprotein 1 (STIP1) was initially identified as an auxiliary partner for heat shock proteins (HSPs) 70 and 90 to modulate the function of HSPs by modulating their dimer structure (11). In addition to its role as a scaffold protein, STIP1 was also found to have a pivotal role in regulating transcription and intracellular signaling transduction, as well as cell proliferation and division (12–14). Consistently, accumulating evidences show that STIP1 is involved in several critical processes that mediate tumor progression including proliferation, migration, and invasion (15–18), indicating its necessity in the development of cancer. Conventionally, STIP1 was considered as a typical cytoplasmic protein due to its lack of transmembrane domain or trans-signal peptide (11). However, recent studies revealed that STIP1 could be secreted by several types of cancer cells, including HCC, and serves as a cytokine in regulating tumor progression (19–21), which strongly suggests serum STIP1 is a promising circulating biomarker for HCC. However, the clinical significance of serum STIP1 remains largely unknown.

Here, in the present study, we evaluated the prognostic value of pretreatment serum STIP1 and dynamic changes in STIP1 levels in HCC. In addition, we assessed the utility of serum STIP1 detection for predicting TACE response. Importantly, we also investigated the value of pretreatment STIP1 levels for predicting microvascular invasion (MVI).

## Methods and Materials

### Patients

Two independent cohorts of HCC patients were enrolled in the present study. Cohort I was recruited from January 2011 to December 2012 and included 161 HCC patients who received curative resection at Zhongshan Hospital as the resection group. All patients in the resection group were Barcelona Clinic Liver Cancer (BCLC) stage 0 or A. Cohort II was recruited from January to December 2014 and included a total of 179 patients who received TACE as the TACE group. All patients in the TACE group were BCLC stage B. Enrollment criteria were as follows (22): (1) definitive HCC diagnosis; (2) no prior anti-HCC treatment; (3) complete resection of all tumor lesions with the cut surface being free of cancer; (4) TACE treatment targeting intrahepatic lesions; and (5) availability of complete clinicopathological and follow-up data. Exclusion criteria were as follows: (1) Child-Pugh C or severe liver dysfunction; (2) receiving any treatment before enrollment; (3) suffering intrahepatic or extrahepatic metastases; (4) having history of any malignancy other than HCC; and (5) insufficient available data. HCC diagnosis in the resection group was based on histopathology examination, while diagnosis was based on imaging scans according to the American Association for the Study of Liver Disease guidelines in the TACE group (22). MVI was defined according to a previous study and was examined by senior pathologists (23). In addition, a total of 122 HDs and 55 patients with CHB without any sign of malignancy were enrolled as negative controls. Approval for the use of human subjects was obtained from the Research Ethics Committee of Zhongshan Hospital. Importantly, informed consent was obtained from every individual who participated in the study.

### Follow-Up and Prognosis Evaluations

Post-treatment surveillance was performed according to our previous studies. Follow-up ended on December 2018. Time to recurrence (TTR), time to progression (TTP), and overall survival (OS) were set as end points of follow-up in present study (24). TTR was defined as the time interval between resection and intrahepatic recurrence or the date of the last follow-up. TTP was defined as the time interval between TACE and disease progression according to mRECIST or the date of the last follow-up (25). OS was defined as the interval between treatment and the death of any cause or last observation date.

### Sample Collection and STIP1 Level Determination

Serum samples were collected from all patients enrolled at baseline (1 or 2 days before interventions) and 1 month after treatments as we did previously (22). STIP1 concentrations were determined by enzyme-linked immunosorbent assay (ELISA) using the Human STIP1 ELISA Kit (Cat: LS-F7598, LifeSpan, USA), according to the manufacturer's instructions. Samples were 2-fold diluted by PBS to avoid exceeding detection limit (>100 ng/ml). The optimal cutoff values of STIP1 in resection and TACE groups were determined by receiver operating characteristics curve analysis, and were set as 83.43 and 112.06 ng/ml, respectively.

### Evaluation of Single TACE Response

Single TACE response evaluation was conducted 1 month after initial treatment according to our previous study (26). Contrast-enhanced computed tomography (CT) or magnetic resonance imaging (MRI) scans were conducted, and results were interpreted by senior, experienced radiologists in imaging diagnosis. Treatment responses were assessed based on 1.1 modified RECIST and patients were stratified as follows: complete response (CR, *n* = 0); partial response (PR, *n* = 136); stable disease (SD, *n* = 21); and progressive disease (PD, *n* = 22). CR and PR were further defined as objective response (OR) according to previous studies (27, 28).

### Statistical Analysis

Statistical analysis was performed with SPSS 21.0 software (IBM, USA). Continuous variables are shown as mean ± standard error of the mean, and chi-squared test, Fisher's exact probability test, and Student's *t*-tests were conducted to determine significant differences between the groups. If the data were not homogeneous, the non-parametric Mann-Whitney U test was applied. Receiver operating characteristic curve analysis was conducted to assess the value of STIP1 level for predicting response to TACE and MVI presence in HCC. Prognostic values were evaluated via Kaplan-Meier curve analysis, log-rank tests, and Cox proportional hazard ratio models. Logistic regression analysis was performed to evaluate the significance of STIP1 in predicting response to TACE. A *P*-value of < 0.05 was considered statistically significant.

## Results

### Baseline Characteristics of HCC Patients Enrolled

**Figure 1:**
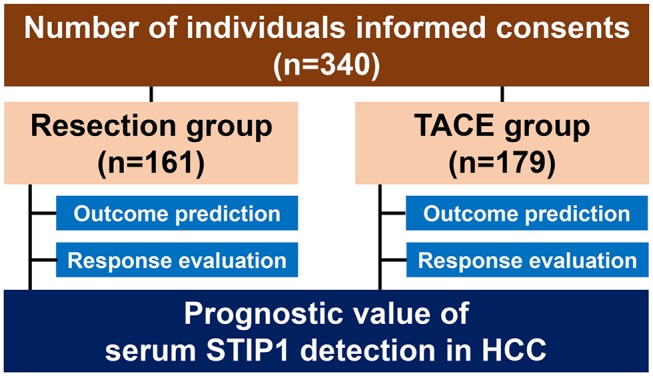
Schematic diagram of present study.

Overall, 517 individuals were recruited in the present study (HCC, 340; chronic hepatitis B, 55; healthy donors, 122, Figure 1). Baseline characteristics are illustrated in Table 1. Mean age was 53.38 years for the resection group and 50.25 years for the TACE group, and 16.15% of patients in the resection group were female while 11.73% of patients in the TACE group were female. HBsAg positivity was 83.85% in the resection group and 89.39% in the TACE group. According to BCLC staging criteria, all patients who underwent resection were BCLC stage 0 or A, while all patients undergoing TACE were BCLC stage B.

**Table 1:** Correlation between clinicopathological parameters of patients enrolled.

**Variables**		**Resection (*****n*** **=** **161)**			**TACE (*****n*** **=** **179)**		
		**STIP1 ≤ 83.43 ng/ml**	**STIP1 > 83.43 ng/ml**	***P***	**STIP1 ≤ 112.06 ng/ml**	**STIP1 > 112.06 ng/ml**	***P***
Sex	Female	10	16	0.365	6	15	0.206
	Male	65	70		68	90	
Age	≤ 50 year	29	36	0.680	30	52	0.235
	>50 year	46	50		44	53	
HBsAg	Negative	13	13	0.924	9	10	0.523
	Positive	62	73		65	95	
ALT	≤ 40 U/L	47	52	0.775	45	61	0.716
	>40 U/L	28	34		29	44	
AFP	≤ 400 ng/ml	60	61	0.184	46	58	0.355
	>400 ng/ml	15	25		28	47	
Cirrhosis	No	3	3	0.864	8	6	0.211
	Yes	72	83		66	99	
Tumor size	≤ 5 cm	60	52	0.012	21	25	0.491
	>5 cm	15	34		53	80	
Number	Single	63	76	0.420	Not applicable		
	Multiple	12	10				
MVI	Absent	57	38	<0.001	Not applicable		
	Present	18	48				
Encapsulation	Complete	60	51	0.005	Not applicable		
	Incomplete	15	35				
Differentiation	I–II	52	52	0.240	Not applicable		
	III–IV	23	34				

*HR, hazard ratio; HBsAg, hepatitis B surface antigen; ALT, alanine aminotransferase; AFP, α-fetoprotein; STIP1, Stress Induced Phosphoprotein 1; MVI, microvascular invasion*.

### Serum STIP1 Was Elevated in HCC and Associated With Tumor Progression

**Figure 2:**
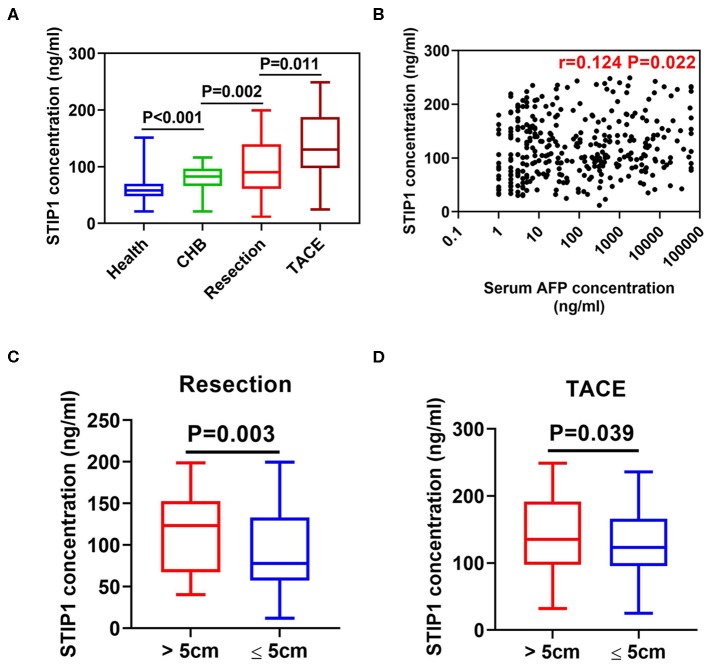
Serum STIP1 was elevated in HCC and associated with tumor progression. **(A)** Distributions of serum STIP1 in HCC patients received curative resection (*n* = 161) and TACE (*n* = 179). Health donors (*n* = 122) and CHB patients (*n* = 55) was enrolled as controls. **(B)** Correlation between baseline serum STIP1 level and baseline serum AFP level. **(C)** Distribution of serum STIP1 of patients with distinct tumor size in resection group. **(D)** Distribution of serum STIP1 of patients with distinct tumor size in TACE group.

Intermediate-HCC patients who received TACE showed the highest serum STIP1 levels, while early patients undergoing curative resection also had significantly higher STIP1 levels than CHB (*P* = 0.002) and HD (*P* < 0.001) individuals (Figure 2A). Interestingly, CHB patients also exhibited higher STIP1 levels than HDs. Further investigation indicated STIP1 levels showed a weak correlation with AFP level (*r* = 0.124, *P* = 0.022; Figure 2B). Moreover, we found patients with bigger tumor sizes (diameter over 5 cm) exhibited significantly higher serum STIP1 concentrations in both the resection (*P* = 0.003; Figure 2C) and TACE (*P* = 0.039; Figure 2D) groups. Together, our data demonstrated that serum STIP1 levels were elevated in HCC patients, and a higher concentration might indicate HCC progression.

### Determination of Cutoff Value of Pretreatment Serum STIP1 for Predicting Prognosis in Resection and TACE Group

**Figure 3:**
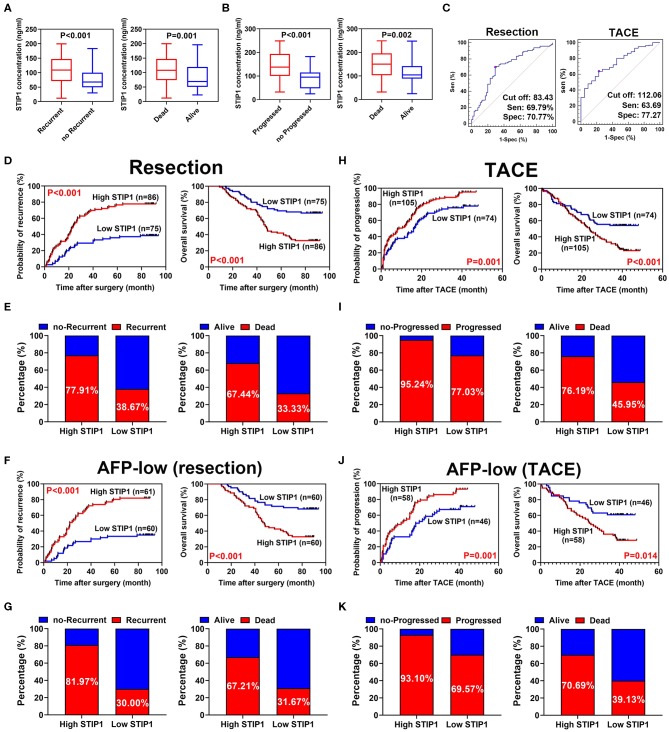
Prognostic significance of pretreatment STIP1 in resectable and irresectable HCC. **(A)** Pretreatment STIP1 levels were significantly elevated in patients suffered recurrence or death in resection group. **(B)** Pretreatment STIP1 levels were significantly elevated in patients suffered progression or death in TACE group. **(C)** ROC curve analyses were conducted to determine the optimal cutoff values for patients received resection or TACE, respectively, via calculating Youden index. **(D)** Kaplan-Meier analyses of TTR (left) and OS (right) according to pretreatment STIP1 level in patients received curative resection. **(E)** Recurrence (left) and death (right) rates of patients with distinct pretreatment STIP1 levels in patients received curative resection. **(F)** Kaplan-Meier analyses of TTR (left) and OS (right) according to pretreatment STIP1 level in low-AFP (≤ 400 ng/ml) patients received curative resection. **(G)** Recurrence (left) and death (right) rates of patients with distinct pretreatment STIP1 levels in low-AFP patients received curative resection. **(H)** Kaplan-Meier analyses of TTP (left) and OS (right) according to pretreatment STIP1 level in patients received TACE. **(I)** Progression (left) and death (right) rates of patients with distinct pretreatment STIP1 levels in patients received TACE. **(J)** Kaplan-Meier analyses of TTP (left) and OS (right) according to pretreatment STIP1 level in low-AFP patients received TACE. **(K)** Progression (left) and death (right) rates of patients with distinct pretreatment STIP1 levels in low-AFP patients received TACE.

Pretreatment STIP1 levels were significantly higher in patients who encountered recurrence (*P* < 0.001) or death (*P* = 0.001, Figure 3A) in the resection group. Similarly, patients who experienced progression (*P* < 0.001) or death (*P* = 0.002) also exhibited significantly higher pretreatment STIP1 levels (Figure 3B). The above results indicated pretreatment STIP1 might act as a powerful tool for predicting prognosis in HCC. Because patients in the TACE group had significantly higher pretreatment STIP1 levels than the patients in the resection group, an independent cutoff value was set for these two groups to achieve the satisfactory performance of STIP1. Using receiver operating characteristic (ROC) curve analysis, 83.43 ng/ml was found to harbor the biggest Youden index when predicting recurrence in the resection group (sensitivity, 69.79%; specificity, 70.77%; Figure 3C), whereas 112.06 ng/ml was found to obtain the biggest Youden index when predicting progression in the TACE group (sensitivity, 63.69%; specificity, 77.27%). Therefore, these two values were set as cutoff values to stratify HCC patients into different pretreatment STIP1 states for subsequent investigations.

### Prognostic Value of Pretreatment STIP1 Level in Resectable HCC

In the resection group, the median follow-up was 36.83 months (range, 1.0–94.0) for TTR and 68.45 months (range, 9.0–94.0) for OS. Patients with high pretreatment STIP1 (>83.43 ng/ml) had significantly shorter TTR (23.60 months vs. not reached, *P* < 0.001) and OS (47.33 months vs. not reached, *P* < 0.001) than patients with low pretreatment STIP1 (≤ 83.43 ng/ml) (Figure 3D). Consistently, patients with high pretreatment STIP1 had higher recurrence (77.91 vs. 38.67%) and death (67.44 vs. 33.33%) rates (Figure 3E). Cox regression analysis revealed that high pretreatment STIP1 level was an independent indicator for both TTR (hazard ratio [HR] 2.57, 95% CI 1.58–4.17, *P* < 0.001) and OS (HR 2.48, 95% CI 1.46–4.20, *P* = 0.001; Tables 2, 3). Moreover, in patients with low AFP levels (≤ 400 ng/ml), STIP1 was significantly correlated with both shorter TTR and OS (both *P* < 0.001; Figure 3F). Also, patients with high pretreatment STIP1 levels had higher recurrence (81.97 vs. 30.00%) and death (67.21 vs. 31.67%) rates in the low-AFP subgroup (Figure 3G). Pretreatment STIP1 retained its prognostic value in conventional low-risk subgroups such as single tumors, small tumor size, well differentiation, or complete encapsulation (all *P* < 0.050; Figure S1).

**Table 2:** Univariate Cox proportional regression analysis of factors associated with recurrence and overall survival after curative resection.

**Variables**	**Recurrence**		**Overall survival**	
	**HR (95% CI)**	***P***	**HR (95% CI)**	***P***
Gender (male vs. female)	1.15 (0.66–1.99)	0.627	1.00 (0.56–1.78)	0.994
Age (>50 vs. ≤50 years)	0.82 (0.55–1.22)	0.319	0.85 (0.55–1.32)	0.471
HBsAg (positive vs. negative)	1.79 (0.99–3.21)	0.050	1.84 (0.97–3.50)	0.064
Cirrhosis (yes vs. no)	5.45 (0.76–39.13)	0.092	4.50 (0.63–32.34)	0.135
ALT (>40 vs. ≤40 U/L)	1.39 (0.91–2.14)	0.128	1.36 (0.87–2.10)	0.171
AFP (>400 vs. ≤400 ng/ml)	1.15 (0.73–1.81)	0.550	1.28 (0.79–2.07)	0.313
Tumor size (>5 vs. ≤5 cm)	1.04 (0.67–1.60)	0.871	0.94 (0.59–1.51)	0.803
Tumor number (multiple vs. single)	4.01 (2/48–6.53)	<0.001	3.71 (2.19–6.27)	<0.001
Microvascular invasion (present vs. absent)	4.59 (3.00–7.03)	<0.001	4.12 (2.60–6.53)	<0.001
Tumor encapsulation (incomplete vs. complete)	2.86 (1.92–4.29)	<0.001	2.24 (1.45–3.47)	<0.001
Tumor differentiation (III–IV vs. I–II)	2.30 (1.54–3.44)	<0.001	2.20 (1.43–3.39)	<0.001
Pretreatment serum STIP1 (>83.43 vs. ≤83.43 ng/ml)	2.97 (1.91–4.60)	<0.001	2.60 (1.62–4.16)	<0.001

*HR, hazard ratio; HBsAg, hepatitis B surface antigen; ALT, alanine aminotransferase; AFP, α-fetoprotein; STIP1, Stress Induced Phosphoprotein 1*.

**Table 3:** Multivariate Cox proportional regression analysis of factors associated with recurrence and overall survival after curative resection.

**Variables**	**Recurrence**		**Overall survival**	
	**HR (95% CI)**	***P***	**HR (95% CI)**	***P***
Tumor number (multiple vs. single)	3.32 (1.92–5.72)	<0.001	2.45 (1.40–4.28)	0.002
Microvascular invasion (present vs. absent)	2.38 (1.49–3.80)	<0.001	1.91 (1.16—.13)	0.011
Tumor encapsulation (incomplete vs. complete)	1.43 (0.91–2.25)	0.123	1.19 (0.74–1.91)	0.483
Tumor differentiation (III–IV vs. I–II)	1.40 (0.91–2.17)	0.127	1.43 (0.90–2.27)	0.128
Pretreatment serum STIP1 (>83.43 vs. ≤83.43 ng/ml)	2.57 (1.58–4.17)	<0.001	2.48 (1.46–4.20)	0.001

*HR, hazard ratio; HBsAg, hepatitis B surface antigen; ALT, alanine aminotransferase; AFP, α-fetoprotein; STIP1, Stress Induced Phosphoprotein 1*.

### Prognostic Value of Pretreatment STIP1 Level in Irresectable HCC

In the TACE group, patients with high pretreatment STIP1 levels (>112.06 ng/ml) had significantly shorter TTP (median 8.23 vs. 16.27 months, *P* = 0.001) and OS (median 23.70 months vs. not reached, *P* < 0.001) than those who had low pretreatment STIP1 levels (≤ 112.06 ng/ml) (Figure 3H). In addition, patients with high pretreatment STIP1 had higher progression (95.24 vs. 77.03%) and death rates (76.19 vs. 45.85%; Figure 3I). Cox regression analysis revealed that high pretreatment STIP1 level was an independent indicator for both TTP (HR 1.61, 95% CI 1.16–2.24, *P* = 0.005) and OS (HR 1.88, 95% CI 1.25–2.82, *P* = 0.002; Tables 4, 5) in the TACE group. In addition, in patients with low AFP levels (≤ 400 ng/ml), STIP1 was significantly correlated with both shorter TTP (*P* = 0.001) and OS (*P* = 0.014; Figure 3J). Also, patients with high pretreatment STIP1 levels had higher progression (93.10 vs. 69.57%) and death (70.69 vs. 39.13%) rates in the low-AFP subgroup (Figure 3K). Additionally, high pretreatment STIP1 was significantly associated with MVI (*P* < 0.001) and incomplete tumor encapsulation (*P* = 0.005; Table 1).

**Table 4:** Univariate Cox proportional regression analysis of factors associated with recurrence and overall survival after TACE.

**Variables**	**Progression**		**Overall survival**	
	**HR (95% CI)**	***P***	**HR (95% CI)**	***P***
Gender (male vs. female)	0.97 (0.60–1.55)	0.887	1.25 (0.68–2.27)	0.473
Age (>50 vs. ≤50 years)	0.79 (0.57–1.08)	0.134	0.65 (0.45–0.94)	0.023
HBsAg (positive vs. negative)	1.23 (0.76–2.00)	0.398	1.85 (0.93–3.70)	0.080
Cirrhosis (yes vs. no)	1.23 (0.68–2.21)	0.499	1.99 (0.88–4.54)	0.100
ALT (>40 vs. ≤40 U/L)	0.96 (0.70–1.32)	0.804	0.97 (0.66–1.41)	0.857
AFP (>400 vs. ≤400 ng/ml)	1.54 (1.12–2.11)	0.008	1.52 (1.05–2.20)	0.026
Tumor size (>5 vs. ≤5 cm)	1.25 (0.87–1.80)	0.231	1.18 (0.77–1.80)	0.456
Pretreatment serum STIP1 (>112.06 vs. ≤112.06 ng/ml)	1.69 (1.22–2.35)	0.002	1.95 (1.30–2.91)	0.001

*HR, hazard ratio; HBsAg, hepatitis B surface antigen; ALT, alanine aminotransferase; AFP, α-fetoprotein; STIP1, Stress Induced Phosphoprotein 1*.

**Table 5:** Multivariate Cox proportional regression analysis of factors associated with progression and overall survival after TACE.

**Variables**	**Progression**		**Overall survival**	
	**HR (95% CI)**	***P***	**HR (95% CI)**	***P***
Age (>50 vs. ≤50 years)	Not applicable		0.74 (0.51–1.08)	0.116
AFP (>400 vs. ≤400 ng/ml)	1.44 (1.05–1.98)	0.025	1.46 (1.10–2.12)	0.046
Pretreatment serum STIP1 (>112.06 vs. ≤112.06 ng/ml)	1.61 (1.16–2.24)	0.005	1.88 (1.25–2.82)	0.002

*HR, hazard ratio; HBsAg, hepatitis B surface antigen; ALT, alanine aminotransferase; AFP, α-fetoprotein; STIP1, Stress Induced Phosphoprotein 1*.

### Prognostic Value of Dynamic Changes of Serum STIP1 in Patients With Resectable HCC

**Figure 4:**
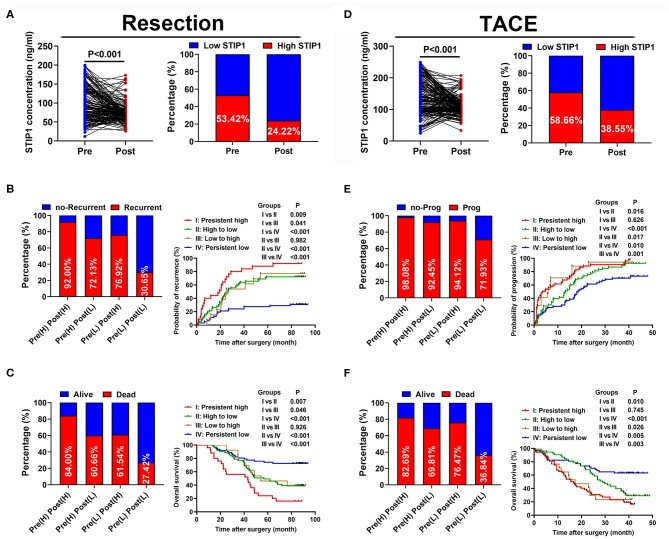
Prognostic values of dynamic changes of serum STIP1 in patients with HCC. **(A)** Distribution of serum STIP1 level (left) and proportion of high STIP1 (right) during perioperative period in HCC patients received curative resection. **(B)** Recurrence rates (left) and Kaplan-Meier curve analyses of TTR (right) in HCC patients received curative resection according to their dynamic changes of serum STIP1 level. **(C)** Death rates (left) and Kaplan-Meier curve analyses of OS (right) in HCC patients received curative resection according to their dynamic changes of serum STIP1 level. **(D)** Distribution of serum STIP1 level (left) and proportion of high STIP1 (right) during peri-treatment period in HCC patients received TACE. **(E)** Progression rates (left) and Kaplan-Meier curve analyses of TTP (right) in HCC patients received TACE according to their dynamic changes of serum STIP1 level. **(F)** Death rates (left) and Kaplan-Meier curve analyses of OS (right) in HCC patients received TACE according to their dynamic changes of serum STIP1 level.

We further explored the dynamic changes in STIP1 during the perioperative period in 169 patients who received curative resection. STIP1 levels were significantly decreased 1 month after surgery (*P* < 0.001; Figure 4A). Similarly, the percentage of patients with high STIP1 was also reduced after surgery (53.42 vs. 24.22%). These patients were further divided into four groups based on perioperative STIP1 levels: Group I, both high for pretreatment and post-treatment (*n* = 25); Group II, pretreatment high and post-treatment low (*n* = 61); Group III, pretreatment low and post-treatment high (*n* = 13); and Group IV, both low pretreatment and post-treatment (*n* = 62). Recurrence rates were 92.00, 72.13, 76.92, and 30.05%, respectively (Figure 4B). Median TTR was significantly shorter for Group I than for Group II (*P* = 0.009), Group III (*P* = 0.041), and Group IV (*P* < 0.001). Meanwhile, patients in Group II and Group III also had significantly shorter TTR than patients in Group IV (both *P* < 0.001). No significant difference in TTR between patients in Group II and Group III was observed (*P* = 0.982; Figure 4B). Death rates were 84.00, 60.66, 61.54, and 27.42%, respectively (Figure 4C). Median OS was significantly shorter for Group I than for Group II (*P* < 0.001), Group III (*P* = 0.046), and Group IV (*P* < 0.001). Also, patients in Group II and Group III had significantly shorter OS than patients in Group IV (both *P* < 0.001). No significant difference in OS between patients in Group II and Group III was observed (*P* = 0.926; Figure 4C).

### Prognostic Value of Dynamic Changes of Serum STIP1 in Patients With Irresectable HCC

STIP1 levels showed a significant reduction after TACE (*P* < 0.001) and the percentage of patients with high STIP1 was also decreased after TACE (58.66 vs. 38.55%; Figure 4D). All 179 patients in the TACE group were divided into four groups based on their pre- and post-TACE STIP1 levels. Progression rates were 98.08, 92.45, 94.12, and 71.93% for these four groups, respectively (Figure 4E). Median TTP was significantly shorter in Group I than in Group II (*P* = 0.016) and Group IV (*P* < 0.001), and significantly shorter TTP was observed in Group III when compared with Group II and IV (both *P* < 0.050). Patients in Group II also had significantly shorter TTP than patients in Group IV (*P* = 0.010). Death rates were 82.69, 69.81, 76.47, and 36.84%, respectively. Median OS was significantly shorter in Group I than in Group II (*P* = 0.016) and Group IV (*P* < 0.001), and significantly shorter OS was observed in Group III when compared with Group II and IV (both *P* < 0.050; Figure 4F). Patients in Group II also had significantly shorter OS than patients in Group IV (*P* = 0.005).

### Post-TACE but Not Pre-TACE STIP1 Level as a Promising Marker for Predicting Tumor Response to Single TACE Treatment

**Figure 5:**
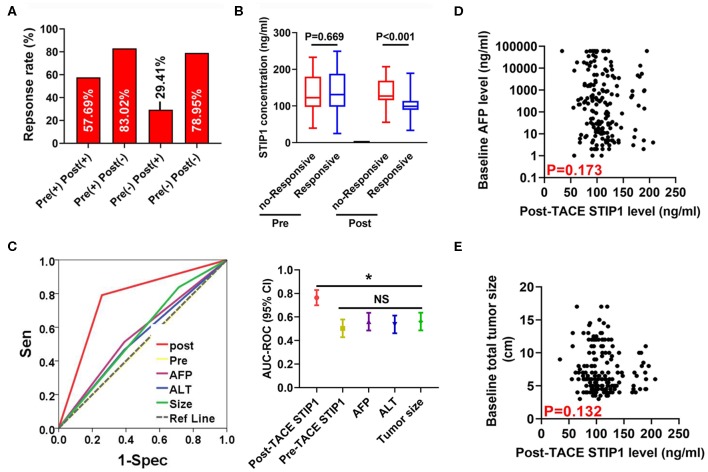
Post-treatment STIP1 level as a novel indicator for predicting objective response after TACE. **(A)** Objective response (defined as CR+PR) rates in HCC patients received TACE according to their dynamic changes of serum STIP1 levels. **(B)** Distributions of pre- and post-TACE serum STIP1 levels in HCC patients with distinct response to TACE. **(C)** ROC curves of different variates for predicting OR after TACE. **(D)** Correlation between baseline AFP levels and post-TACE STIP1 levels. **(E)** Correlation between baseline AFP levels and post-TACE STIP1 levels. *P < 0.05.

We first observed the single TACE response rate (defined as PR+CR according to the definition of objective response rate) in four groups stratified by peri-TACE STIP1 levels. Response rates were 57.69, 83.02, 29.14, and 78.95%, respectively (Figure 5A). Further investigations revealed that pre-TACE STIP1 levels showed no significant difference between responsive and non-responsive patients (*P* = 0.669), whereas post-TACE STIP1 levels were significantly decreased in responsive HCC patients (*P* < 0.001; Figure 5B). Multivariate logistic regression analysis indicated that post-TACE STIP1 level was the most powerful independent indicator for predicting response (odds ratio 21.09, 95% CI 7.37–60.23, *P* < 0.001; Table S1). Consistently, ROC curve analysis demonstrated post-TACE STIP1 had the largest AUC-ROC (AUC = 0.767) for predicting response among all variates investigated (Figure 5C). However, post-TACE STIP1 level showed no correlation with baseline AFP level or baseline total tumor size (both *P* > 0.050; Figures 5D,E).

### Pretreatment STIP1 Level as a Novel Indicator for Predicting MVI in HCC

**Figure 6:**
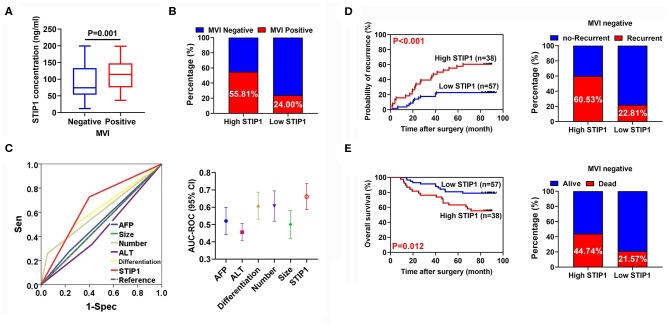
Pretreatment serum STIP1 as a powerful predictor for MVI. **(A)** Comparison of pretreatment STIP1 levels between HCC patients with or without MVI. **(B)** MVI positive rates in HCC patients received curative resection according to pretreatment STIP1 levels. **(C)** ROC curves of various parameters for predicting MVI in HCC patients. **(D)** Kaplan-Meier curve of TTR (left) and recurrence rates (right) according to pretreatment STIP1 level in MVI-negative patients received curative resection. **(E)** Kaplan-Meier curve of OS (left) and recurrence rates (right) according to pretreatment STIP1 level in MVI-negative patients received curative resection.

Among all patients enrolled in the resection group, 66 (40.99%) patients encountered MVI. Pretreatment STIP1 levels were significantly increased in patients with MVI (*P* < 0.001; Figure 6A). Moreover, patients with high STIP1 had higher MVI-positive rates (55.81 vs. 24.00%; Figure 6B). ROC analysis revealed that pretreatment STIP1 exhibited the largest AUC (AUC = 0.644) among all variables explored (Figure 6C and Table S2). However, other involved predictors including AFP (AUC = 0.521), ALT (AUC = 0.456), differentiation (AUC = 0.611), tumor number (AUC = 0.602), and tumor size (AUC = 501) showed unsatisfactory performance. We further investigated the prognostic role of STIP1 in the MVI-absent subgroup. Similarly, pretreatment STIP1 was also significantly correlated with both shorter TTR (*P* < 0.001) and OS (*P* = 0.012; Figure 6D) in patients without MVI. Concordantly, MVI-absent patients with high pretreatment STIP1 levels had higher recurrence (60.53 vs. 22.81%) and death (44.74 vs. 21.57%) rates (Figure 6E).

## Discussion

Despite the great improvements made in last two decades, prognosis of HCC remains unsatisfactory (3). Such an embarrassing situation might be partially attributed to lack of a promising and reliable biomarker for outcome prediction and real-time surveillance of tumor progression. Here, we demonstrated serum STIP1 could serve as a novel biomarker to discriminate HCC patients with high risk of developing progression. Moreover, monitoring peri-treatment dynamic changes of STIP1 could provide useful information for predicting long-term prognosis after treatment. Importantly, post-TACE STIP1 level was identified as a powerful indicator to reflect the response to TACE. Specifically, our data also confirmed the clinical utility of STIP1 detection in predicting MVI.

As a crucial co-chaperone of HSP90 complex, STIP1 was reported to execute its elemental function with HSP90, resulting in rapid cancer progression (11). STIP1 was preferentially expressed in cancerous tissues of various solid tumors, including HCC, and high STIP1 expression was closely associated with dismal outcomes (11, 18, 21, 29). Moreover, functional assays confirmed STIP1 as a vital pro-oncogene during carcinogenesis process (30, 31). Interestingly, recent studies demonstrated STIP1 could be secreted by tumor cells and act as a critical cytokine to regulate malignant phenotype (17). STIP1 has been identified as crucial regulator of HCC progression. Previous studies have demonstrated that STIP1 promoted metastases foci formation via activating snail transcription and subsequently epithelial-to-mesenchymal transition in an HSP-dependent manner (31). Moreover, it could provoke HCC progression via interaction with Axin and DVL2 to activate beta-catenin signaling (29). Of note, secretory form STIP1 could stimulate HCC progression in an autocrine manner (21), which led us to raise the hypothesis that serum STIP1 might be a potential biomarker for predicting prognosis of HCC. Here, we showed that serum STIP levels were significantly elevated in HCC patients compared with either CHB patients or HDs. Intriguingly, STIP1 levels increased with HCC progression, suggesting the potential role of STIP1 in HCC diagnosis. Further investigation demonstrated pretreatment STIP1 level was an independent indicator for both tumor progression and survival, regardless of therapeutic approach. Clinically, AFP is currently the mostly widely used serum biomarker for evaluating prognosis. However, monitoring progression in low-AFP subgroups remains a challenge (22). We found serum STIP1 retained its prognostic value in low-AFP subgroups, suggesting STIP1 might be a useful supplement to AFP detection to achieve more accuracy in identifying patients with dismal outcomes. Together, our data indicated pretreatment STIP1 is a powerful and feasible biomarker for predicting prognosis in HCC.

Monitoring dynamic changes in tumor biomarkers during the peri-treatment period could provide critical information to reflect the disease status after treatment. Our data indicated serum STIP1 levels were dramatically decreased after treatment, and patients whose STIP1 level remained high or became high after treatment suffered significantly worse long-term prognosis in both the resection and TACE groups. Because tumors were completely removed in patients who underwent curative resection, dynamic changes in STIP1 might reflect the micro-dissemination that could not be observed during operation. Thus, perioperative dynamic changes in STIP1 might be a valuable basis for the application of adjuvant interventions such as TACE or sorafenib after surgery. Meanwhile, for patients who received TACE, the predictive value of monitoring peri-TACE dynamic changes in STIP1 for long-term survival might reflect the intrinsic characteristics of HCC toward hypoxia and cytotoxicity. Thus, more importantly, our findings provide a more powerful and reliable basis to precisely predict the outcomes after TACE, as evidenced by greater AUC-ROC (Figure S2).

TACE is recommended as the first therapeutic approach for intermediate-HCC patients by BCLC criteria (32). However, heterogeneous responses to TACE were widely observed in clinical practice (33). Unfortunately, conventional pathological parameters for predicting treatment response were not available for patients in this study who received TACE due to difficulties in obtaining biopsy samples. Meanwhile, serum biomarkers are considered an ideal tool to monitor treatment response to TACE with the advantages of easy acquisition and noninvasiveness. Moreover, serum detection provides a safer and more convenient approach than imaging scans with the advantage of non-exposure to radiation. Here, we found post-TACE but not pre-TACE STIP1 levels were closely associated with the objective response rate after single TACE treatment. Further ROC curve analysis demonstrated the satisfactory performance of post-TACE STIP1 level for predicting OR. Notably, post-TACE STIP1 level showed no correlation with baseline tumor size or AFP level, suggesting the universal use of post-TACE STIP1 level in evaluating response to single TACE treatment, regardless of baseline HCC status. Together, our data indicated that post-TACE STIP1 detection was a promising non-invasive method with strong predictive power toward therapeutic response.

Accumulating clinical evidence confirmed the presence of MVI as a significant risk indicator for worse outcomes in HCC (34). Clinically, the presence of MVI acts as a crucial indicator for selecting appropriate therapeutic intervention for HCC patients (35). Unfortunately, most of the risk factors associated with MVI are pathological characteristics that can only be determined in resected samples or biopsy, posing a problem in routine clinical practice (36, 37). Therefore, identification of serum biomarkers for predicting MVI before treatment might improve HCC management. Here, we reported that patients with high pretreatment serum STIP1 levels had a high possibility of harboring MVI, and high STIP1 was confirmed as an independent predictor for MVI. Moreover, we found the predictive performance of STIP1 was stronger than other biomarkers such as AFP, tumor size, and tumor number. Thus, our findings demonstrate a powerful tool for providing accurate and useful information for MVI prediction in HCC, enabling early clinical decisions to tailor appropriate therapeutic approaches for individualized therapy in HCC.

There are several limitations in our present study. First, it was a single-centered, retrospective study. Therefore, prospective and external validations are further needed in the future. Second, most patients enrolled had HBV backgrounds, which greatly differed from the patients in United States or Europe (38). We could not exclude the possibility that the predictive value of serum STIP1 was not applicable in HCC patients with other etiology backgrounds, and further confirmation is also needed. Finally, despite the satisfactory performance of STIP1, more investigations should be conducted to optimize the best cutoff or generate novel index containing STIP1 and other serum biomarkers to improve the discrimination power.

## Conclusions

In summary, our data demonstrated the prognostic significance of serum STIP1 in HCC. Importantly, dynamic changes in STIP1 were found to exert great significance in reflecting treatment response, especially in predicting objective response to single TACE intervention. Moreover, our data indicated STIP1 detection as a useful tool for predicting MVI before surgery with the advantages of convenience and accuracy. Integration of serum STIP1 detection into HCC management might facilitate early clinical decision-making to improve the prognosis of HCC patients in the future.

## Data Availability Statement

The raw data supporting the conclusions of this article will be made available by the authors, without undue reservation, to any qualified researcher.

## Ethics Statement

The studies involving human participants were reviewed and approved by Research Ethics Committee of Zhongshan Hospital. The patients/participants provided their written informed consent to participate in this study.

## Author Contributions

X-LM, GL, and R-QL: conceptualization. X-LM, S-HX, M-JY, M-LW, and W-GT: methodology. GL and R-QL: validation, visualization, supervision, and project administration. X-LM, M-LW, and M-JY: formal analysis. X-LM and M-JY: investigation. W-GT, GL, and R-QL: resources. X-LM, GL, and R-QL: data curation. X-LM: writing—original draft preparation. All authors: writing (review and editing). W-GT, GL, and R-QL: funding acquisition.

## Conflict of Interest

The authors declare that the research was conducted in the absence of any commercial or financial relationships that could be construed as a potential conflict of interest. The reviewer JZ declared a shared affiliation, with no collaboration, with the authors to the handling editor at the time of review.

## Abbreviations

STIP1: stress-induced-phosphoprotein 1
HCC: hepatocellular carcinoma
TACE: transcatheter arterial chemoembolization
ELISA: enzyme-linked immunosorbent assay
ROC: receiver operating characteristic
OR: objective response
CHB: chronic hepatitis B
MVI: microvascular invasion
OS: overall survival
TTR: time to recurrence
TTP: time to progression
BCLC: Barcelona Clinic Liver Cancer
HSP: heat shock protein
CT: computed tomography
MRI: magnetic resonance imaging
CR: complete response
PR: partial response
SD: stable disease
PD: progressive disease.
