# Supplementary material for: Serum STIP1, a Novel Indicator for Microvascular Invasion, Predicts Outcomes and Treatment Response in Hepatocellular Carcinoma
Source: Front Oncol. 2020 Apr 30;10:511. doi: 10.3389/fonc.2020.00511 (PMC7212360; doi:10.3389/fonc.2020.00511)
Supplement: Supplementary file 1 [file Data_Sheet_1.docx]

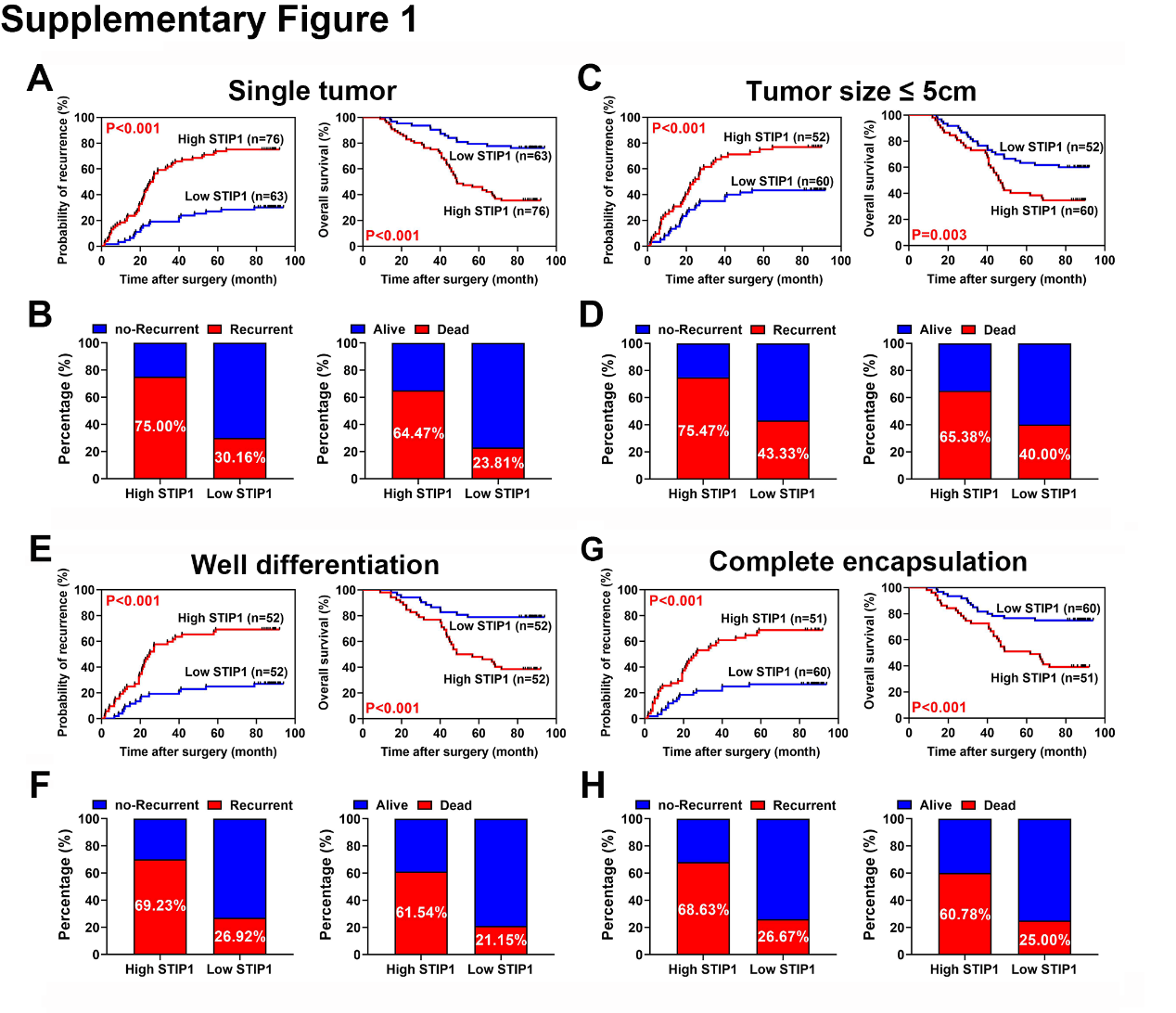


**Figure S1**. Prognostic significance of pretreatment STIP1 level in conventional low-risk subgroups. (A) Kaplan-Meier curve analyses of TTR (left) and OS (right) according to pretreatment STIP1 level in patients who had only one tumor lesion. (B) Recurrence (left) and death (right) rates of patients with distinct pretreatment STIP1 levels in patients who had only one tumor lesion. (C) Kaplan-Meier curve analyses of TTR (left) and OS (right) according to pretreatment STIP1 level in patients with small HCC (total diameter≤5 cm). (D) Recurrence (left) and death (right) rates of patients with distinct pretreatment STIP1 levels in patients with small HCC. (E) Kaplan-Meier curve analyses of TTR (left) and OS (right) according to pretreatment STIP1 level in patients with well differentiation HCC. (F) Recurrence (left) and death (right) rates of patients with distinct pretreatment STIP1 levels in patients with well differentiation HCC. (G) Kaplan-Meier curve analyses of TTR (left) and OS (right) according to pretreatment STIP1 level in patients with complete encapsulation. (H) Recurrence (left) and death (right) rates of patients with distinct pretreatment STIP1 levels in patients with complete encapsulation.


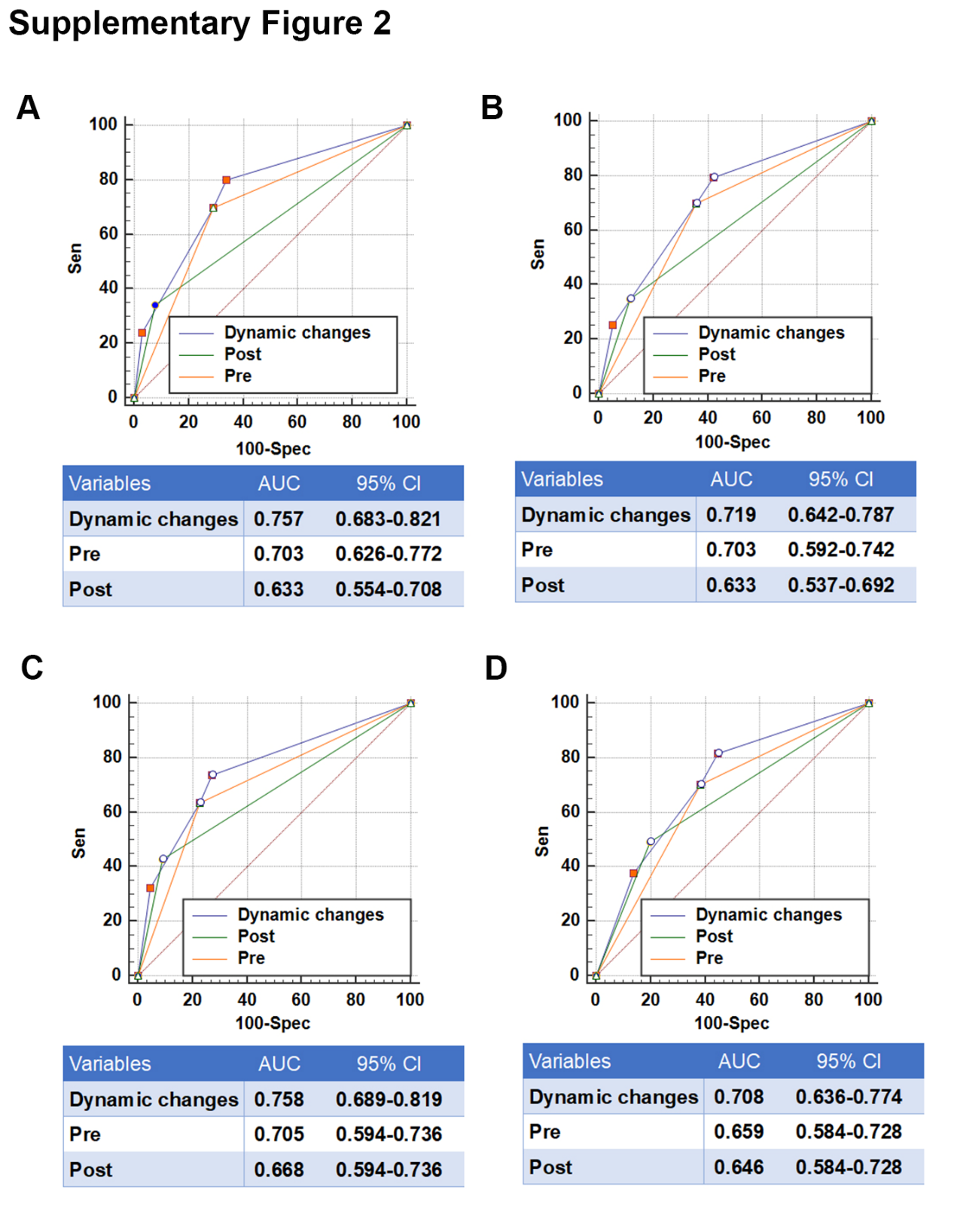


**Figure S2**. ROC curves of pretreatment, posttreatment, and dynamic changes of serum STIP1 for predicting prognosis in HCC. (A) ROC curves of pretreatment, posttreatment, and dynamic changes of serum STIP1 for predicting TTR in patients received curative resection. (B) ROC curves of pretreatment, posttreatment, and dynamic changes of serum STIP1 for predicting OS in patients received curative resection. (C) ROC curves of pretreatment, posttreatment, and dynamic changes of serum STIP1 for predicting TTP in patients received TACE. (D) ROC curves of pretreatment, posttreatment, and dynamic changes of serum STIP1 for predicting OS in patients received TACE.

| **Table S1.** Multivariate logistic regression analysis of factors associated with OR in TACE group | | |
| --- | --- | --- |
| Variables | MVI | |
|  | HR  (95% CI) | P |
| Post-TACE serum STIP1  (>112.06 ng/ml versus ≤112.06 ng/ml) | 21.08  (7.37-60.23) | <0.001 |
| Tumor size  (>5 cm versus ≤5 cm) | 3.77  (1.27-11.21) | 0.023 |
| Abbreviations: HR, hazard ratio; STIP1, Stress Induced Phosphoprotein 1 | | |

| **Table S2.** Multivariate logistic regression analysis of factors associated with MVI | | |
| --- | --- | --- |
| Variables | MVI | |
|  | HR  (95% CI) | P |
| Tumor number  (Multiple versus single) | 5.34  (2.44-11.69) | <0.001 |
| Tumor differentiation  (III-IV versus I-II) | 2.47  (1.18-5.20) | 0.017 |
| Pretreatment serum STIP1  (>83.43 ng/ml versus ≤83.43 ng/ml) | 9.69  (2.96-31.78) | <0.001 |
| Abbreviations: HR, hazard ratio; HBsAg, hepatitis B surface antigen; ALT, alanine aminotransferase; AFP, α-fetoprotein; STIP1, Stress Induced Phosphoprotein 1 | | |
